# Effects of Flower and Fruit Extracts of *Melastoma malabathricum* Linn. on Growth of Pathogenic Bacteria: *Listeria monocytogenes, Staphylococcus aureus, Escherichia coli,* and *Salmonella typhimurium*

**DOI:** 10.1155/2013/459089

**Published:** 2013-04-10

**Authors:** Siti Nurhadis Che Omar, Janna Ong Abdullah, Khairul Anuar Khairoji, Sieo Chin Chin, Muhajir Hamid

**Affiliations:** ^1^Department of Microbiology, Faculty of Biotechnology and Biomolecular Sciences, Universiti Putra Malaysia, 43400 Serdang, Selangor, Malaysia; ^2^Institute of Bioscience, Universiti Putra Malaysia, 43400 Serdang, Selangor, Malaysia

## Abstract

*Melastoma malabathricum* Linn. is a shrub that comes with beautiful pink or purple flowers and has berries-like fruits rich in anthocyanins. This study was carried out with the aim to evaluate the inhibitory activities of different concentrations of the *M. malabathricum* Linn. flower and fruit crude extracts against *Listeria monocytogenes* IMR L55, *Staphylococcus aureus* IMR S244, *Escherichia coli* IMR E30, and *Salmonella typhimurium* IMR S100 using the disc diffusion method. The lowest concentrations of the extracts producing inhibition zones against the test microorganisms were used to determine their minimum inhibitory concentrations (MICs) and minimum bactericidal concentrations (MBCs). In addition, the growth of *Listeria monocytogenes* IMR L55 and *Staphylococcus aureus* IMR S244 grown in medium supplemented with the respective extracts at different temperatures (4°C, 25°C, and 37°C) and pHs (4, 6, 7, and 8) was determined.

## 1. Introduction

Antibiotic resistance is currently the greatest challenge to effective treatment of infections globally. The continuous emergence of new antibiotic resistant strains day by day has become a major problem for patients. Microorganisms are endowed with the ability to protect themselves against natural antibiotics by showing resistance through the exchange of genetic materials with other organisms. In a recent study, 25% of bacterial pneumonia cases were shown to be resistant to penicillin, and an additional 25% of cases were resistant to more than one antibiotic [1]. Hence, with the increase of microbial resistance to antibiotics, there is considerable interest in investigating the antimicrobial effects of different types of plant extracts as potential sources of natural antimicrobials against a wide range of microorganisms. 

 Currently, there is a growing interest to use plant extracts of herbs and spices for the preservation of foods, as they possess a characteristic flavour and sometimes show antioxidant and antimicrobial activities [2, 3]. Crude extracts from plants with a history of use in folk medicines have been screened *in vitro* for antibacterial activity by many research groups [4]. 

The sensitivity of a microorganism towards an antimicrobial agent can be tested using the antimicrobial susceptibility test. Conventionally, antimicrobial test results are reported qualitatively and/or quantitatively. Qualitative results are often reported as susceptible (S), intermediate (I), or resistant (R), while quantitative results are reported as minimal inhibitory concentration (MIC), the lowest concentration of the agent in completely inhibiting the growth of the microorganism [5].

The two basic procedures of the antimicrobial susceptibility test are the disc diffusion and broth dilution assays [6]. The disc diffusion assay is based on the diffusion of an antimicrobial agent from the disc placed on the agar surface of a growth medium that has been swabbed with cultured bacteria. Inhibition, which is a measure of activity, is indicated by a zone of no growth around the disc. The broth dilution method involves making a series of dilutions of the agent in the broth use to grow the microorganism, in which failure to grow indicates MIC. The disc diffusion test is often preferred and is commonly used because of its flexibility in the types and numbers of agents that can be tested at one time, and it is more economical [7].

*Melastoma malabathricum* Linn. is a shrub that belongs to the Melastomataceae family. It is found easily along roadsides throughout Malaysia. The flowers have a cup-shaped green calyx with five narrow reddish sepals and five purplish petals. The fruits are berry-like and they break open irregularly, bearing numerous nonendospermous seeds [8, 9] surrounded with purplish pulps. In folk medicines, the leaves of the plant have been used for the treatment of several diseases such as dysentery, diarrhea, scar prevention [10–13], and as anti-infection [12]. Some used the crude extract from the shoots and roots as aspirin to relief toothaches and treat leucorrhea [13]. The young shoots are eaten raw and said to be able to treat high blood pressure and diabetes while the roots are given to postpartum women to aid healing and womb strengthening [14, 15]. 

*M. malabathricum* Linn. petals and fruits are rich sources of flavonoid compounds, such as anthocyanins [9, 16, 17]. Anthocyanins are colouring pigments in flowering plants and they possess antioxidant, phytoalexin, and antibacterial activities [18]. Besides being beneficial to health, anthocyanins also have potential as natural food colourant.

Although antimicrobial actions of flavonoids have been studied extensively, very few researches have been made on antimicrobial activities of *Melastoma* species especially on the flowers and fruits. Hence, in this study we report the antibacterial activity of the methanolic crude flower and fruit extracts of *M. malabathricum* Linn. against *L. monocytogenes* IMR L55, *S. aureus* IMR S244, *E. coli* IMR E30, and *S*. *typhimurium* IMR S100. In addition, the temperature and pH effects on the bacteria growth when subjected to these extracts were also investigated.

## 2. Materials and Methods

### 2.1. Plant Materials

Flowers and fruits of *Melastoma malabathricum* Linn. were collected from wild grown plants found along the Lebuh Silikon road sides in Universiti Putra Malaysia, Serdang, Selangor, Malaysia (latitude: 2.996664°). The samples were collected between 10 and 11 a.m. Authentication of the plant was done at the Institute of Bioscience, Universiti Putra Malaysia, where the voucher specimen was conserved under the reference number SK1517/07.

### 2.2. Extraction of Crude Fruit and Flower Extracts

About 100 g of each fresh petals and fruits was extracted separately overnight with 1000 mL of methanol (Merck, Darmstadt, Germany), at 25 ± 1°C. The extracts were ducked through Whatman no. 1 filter paper (Whatman, Maidstone, England), before concentrated and then vacuum dried in a rotary evaporator (Buchi, Switzerland) at 37 ± 1°C. All steps were carried out in dark condition.

### 2.3. Microbial Strains

The human origin strains of *L. monocytogenes* IMR L55, *S. aureus *IMR S244, *E. coli *IMR E30, and *S. typhimurium* IMR S100 were obtained from the Institute of Medical Research (IMR), Malaysia.

### 2.4. Antibacterial Assays

#### 2.4.1. Preparation of Flower and Fruit Crude Extracts

Concentrated crude flower and fruit extracts of the following amounts: 600 mg, 500 mg, 400 mg, 300 mg, 200 mg, and 100 mg were, respectively, dissolved in 1 mL of water. Then, all the dissolved extracts were separately filtered through a 0.22 *μ*m membrane. A total of 10 *μ*L of each sample extracts was loaded onto each sterile 5 mm diameter paper discs. Sterilized water was used as a negative control. All impregnated discs were allowed to dry overnight at room temperature in a laminar flow hood. Commercial Tetracycline 30 (Oxoid, Hampshire, England) disc was used as a positive control.

#### 2.4.2. Disc Diffusion Assay

The antimicrobial activity was measured by the disc diffusion method [16]. All bacteria were grown for 18–24 h at 37°C in nutrient broth (Merck, Darmstadt, Germany) and the cultures were adjusted to match 0.5 McFarland standard prior to each assay. Petri dishes containing nutrient agar (Merck, Darmstadt, Germany) were swabbed with 100 *μ*L of the microbial suspensions. Discs were impregnated with 10 *μ*L each of 100 mg/mL, 200 mg/mL, 300 mg/mL, 400 mg/mL, 500 mg/mL, and 600 mg/mL extracts and placed on the agar surface. Discs impregnated with water were used as negative controls. The plates were incubated at 37°C for 24 h. All the tests were performed in triplicates and were repeated three times.

#### 2.4.3. Minimum Inhibition Concentration (MIC) and Minimum Bactericidal Concentration (MBC) Determination

The Minimum inhibition concentration (MIC) was determined only for microbial species which showed growth inhibition zone in the disc diffusion assay described above. The bacterial inoculum was prepared from a 18–24 h broth culture using the discs diffusion method described above. A 18–24 h broth culture of each respective microbial species, adjusted to 0.5 McFarland standard, was used as the inoculum in this experiment. Approximately 100 mg each of the crude flower and fruit extracts were dissolved in Nutrient broth separately in a test tube to a final concentration of 100 mg/mL. A two-fold dilution was prepared to give the final concentrations of 0.39, 0.78, 1.56, 3.12, 6.25, 12.5, 25.0, 50.0, and 100.0 mg/mL of the crude flower and fruit extracts. Then, 500 *μ*L (contained approximately 10^6^ to 10^7^ cfu/mL) of the bacterial inoculum was added into each corresponding tube. Crude flower and fruit extracts without bacterial inoculum were used as negative controls. Culture growth was determined macroscopically and recorded after 18–24 h incubation at 37°C. The MIC was determined as the lowest concentration corresponding to the test tube presenting no turbidity observed after incubation.

For the determination of the minimum bactericidal concentration (MBCs), 100 *μ*L of the content from each tested tube in the MIC assay that showed no turbidity changed was used to inoculate onto fresh nutrient agar. The plates were further incubated for 18–24 h at 37°C. The lowest concentration that yielded no growth, at which 99.9% of the bacteria was killed, was determined as the MBC [17]. All tests were performed in triplicates and repeated three times. 

#### 2.4.4. Effects of Temperature and pH

In this study, two bacteria comprised of *L. monocytogenes* strain L55 and *S. aureus* strain S244 were selected to determine the effects of temperature and pH on their growth. These bacteria were selected as they were shown to be significantly inhibited in the extracts based on the disc diffusion, MIC and MBC assays. Approximately 1.0 mL of each extracts was, respectively, added to 9.0 mL of nutrient broth to give a final concentration of 100 mg/mL. The pH of the mixture was adjusted to pHs 4, 6, 7, and 8, respectively, using sterile 1.0 N HCl and 1.0 N NaOH solution. Subsequently, 1 mL (10^6^ to 10^7^ cfu/mL) of *L. monocytogenes* strain L55 and *S. aureus* strain S244 was added, respectively, and the tubes were incubated at 4, 25, and 37°C for 24 h. Aliquots (1 mL) of the cultures were taken at predetermined time intervals (0, 0.5, 1, 2, 4, 8, 12, 16, 20, and 24 hrs) to determine the viable population of the bacteria. At each sampling times, 1 mL of the culture was first serially diluted with saline solution (0.85% NaCl) and then 100 *μ*L aliquot was spread onto nutrient agar plate. The plates were incubated at 37°C for 24 h, and viable colonies were counted using a colony counter. Bacteria cultured in nutrient broth without extract were used as a control. All tests were performed in triplicates and each test was repeated three times [18].

### 2.5. Statistical Analysis

The experiments were repeated three times. The statistical data such as means and standard deviations was performed using ANOVA (SPSS software for Windows, version 17.0). The significance of the differences was determined by Tukey's test at *P* < 0.05 [19].

## 3. Results

### 3.1. Yield of Crude Extracts

 From the extraction procedure, the average yield of the crude flower extract obtained was 6.0 g from a starting 100 g of fresh petals, while the average yield of the crude fruit extract obtained was 9.6 g from 100 g of fresh fruits. Both types of crude extracts were stored at 4°C after being rotary evaporated to concentrate the extracts.

### 3.2. Antibacterial Activities

**Figure 1:**
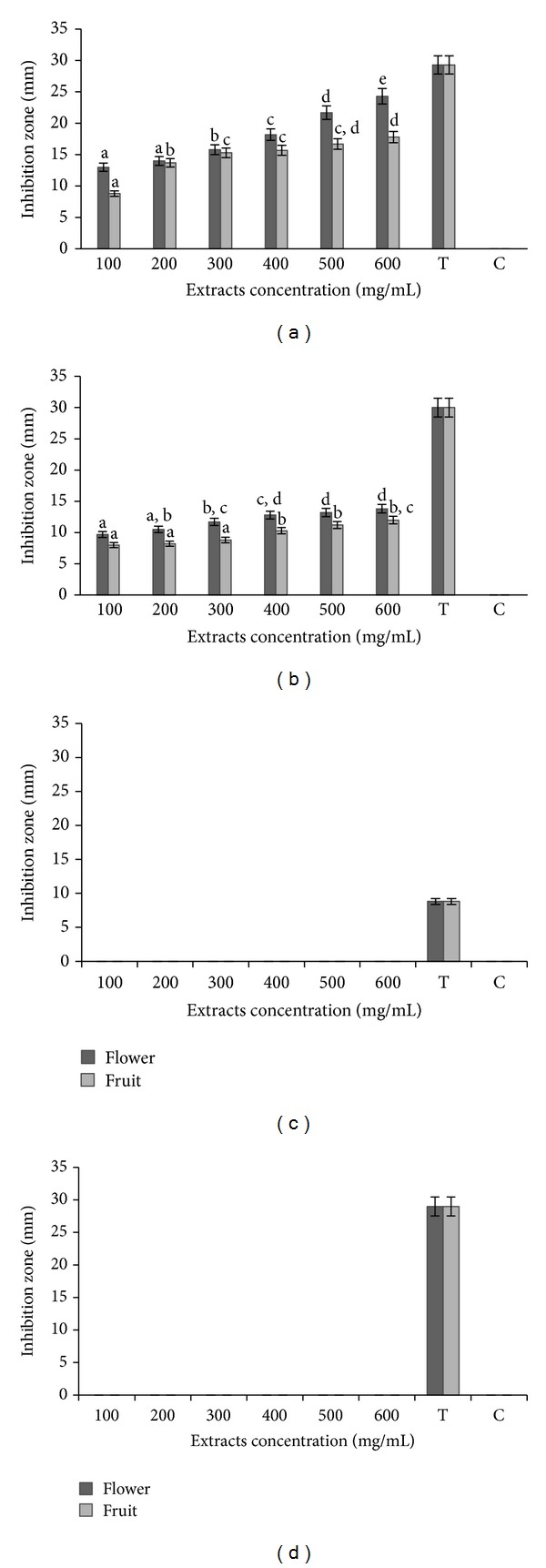
The inhibition zones of flower and fruit extracts of *Melastoma malabathricum* L. against (a) *Listeria monocytogenes* IMR L55, (b) *Staphylococcus aureus* IMR S244, (c) *Escherichia coli *IMR E30, and (d)* Salmonella typhimurium* IMR S100 at different concentrations. (T) Standard antibiotic Tetracycline 30 *µ*g/disc; (C) negative control (water). The values represent the mean ± S.D. of triplicate preformed test for three times repeated. Different letters above each bar indicate significant difference between means (*P* < 0.05) within the same inoculation time.

The crude flower and fruit extracts of *Melastoma malabathricum* Linn. were tested using the disc diffusion method against all bacteria, and the diameter of zone inhibition presented by each discs was recorded as shown in Figure 1. The results were varied based on the concentrations of the extracts used. In this study, the results showed that Gram-positive bacteria were more susceptible to the crude flower and fruit extracts than the Gram-negative species. Both extracts did not exhibit any effects on the *E. coli *IMR E30 (Figure 1(c)) and *S. typhimurium* IMR S100 (Figure 1(d)) in this study. Inhibitory activities of the crude flower and fruit extracts of *M. malabathricum* L. tested using the disc diffusion method against *L. monocytogenes* strain L55 and *S. aureus *strain S244 were summarised in Figures 1(a) and 1(b). The negative control (C) was inactive against the tested bacteria. The flower and crude extracts, at all concentrations (100–600 mg/mL) tested, inhibited the growth of *L. monocytogenes* strain L55 with inhibition zones ranging from 13.0 to 24.3 mm and 8.8 to 17.8 mm, respectively (Figure 1(a)). Both extracts also showed antibacterial activity towards *S. aureus* strain S244 with inhibition zones ranging from 9.7 to 13.8 and 8.0 to 12.0 mm, respectively (Figure 1(b)). The antibacterial effectiveness of both extracts concentrations against both bacteria was as expected in descending sequence with the highest concentration being the most effective. Both bacteria were found to be most sensitive to the flower extract compared to the fruit extract.

The antibacterial activities of both extracts were further confirmed using the microdilution broth assay. The MIC and MBC values were shown in Table 1. The extracts exhibited antibacterial activity against both bacteria and *L. monocytogenes *L55 was more sensitive. The MIC values of 12.5 mg/mL and 100.0 mg/mL were obtained using the flower extract on *L. monocytogenes* L55 and *S. aureus* S244, respectively. For the fruit extract, the MIC values were 12.5 mg/mL and 100.0 mg/mL for *L. monocytogenes* L55 and *S. aureus* S244, respectively. The MBC values of both extracts against the tested bacterial were 100 mg/mL. 

### 3.3. Temperature and pH Effects

Previous work on *M. malabathricum* Linn. flower and fruit crude extracts carried out in the current laboratory revealed that stability of the extracts was affected by pH and temperature [17, 20]. *L. monocytogenes* and *Staphylococcus aureus* are well-known pathogens which have been extensively studied since the first major recognized outbreak in the early 1980s [21]. Hence, in order to explore further the extracts' potential as antibacterials for future use at different pHs and temperatures conditions, the extracts were tested against two types of bacteria, *Listeria monocytogenes* IMR L55 and *Staphylococcus aureus* IMR 244.

**Figure 2:**
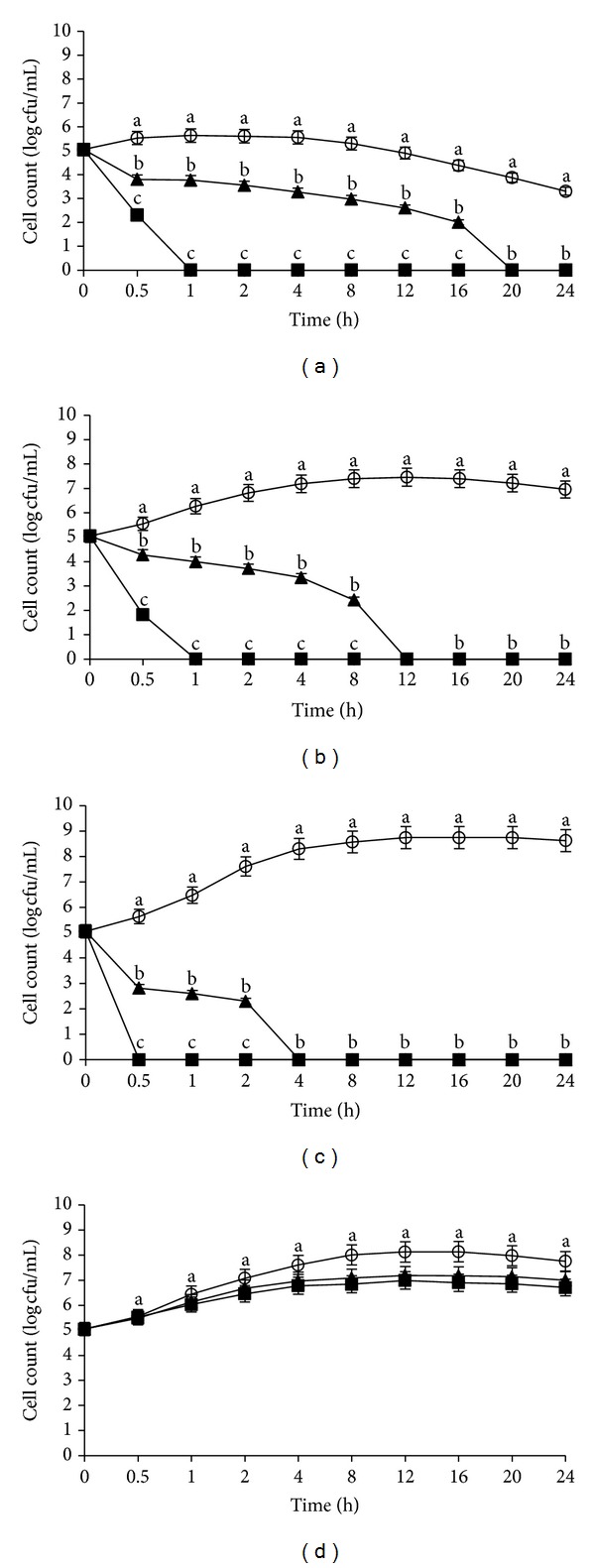
Effects of *Melastoma malabathricum* Linn. extracts on the growth of *Listeria monocytogenes *IMR L55. *L. monocytogenes* IMR L55 was inoculated at various pH values containing 100 mg/mL extracts; (a) pH 4; (b) pH 6; (c) pH 7; (d) pH 8. Cultures were incubated at 37°C. (○) control; (■) flower; (▲) fruit. The values represent the mean ± S.D. of triplicate preformed test for three times repeated. Different letters above each line indicate significant difference between means (*P* < 0.05) within the same inoculation time.

Figure 2 shows the growth profiles of *L. monocytogenes* IMR L55 incubated at 37°C and at different pHs (4, 6, 7, and 8). *L. monocytogenes* IMR L55 could not be recovered after 1 h exposure to the crude flower extract, with a growth inhibition kinetic of 6 log CFU/mL and 6.5 log CFU/mL at pH 4 (Figure 2(a)) and 6 (Figure 2(b)), respectively. Compared to pH 7 (Figure 2(c)), *L. monocytogenes* IMR L55 was completely inactivated with 5.5 log CFU/mL reduction after 30 min of exposure to the extract. At pH 8, there was no significant inhibition observed. Meanwhile, the growth profiles showed that *L. monocytogenes* IMR L55 was less susceptible to the crude fruit extract for all the pHs tested at 37°C. This is seen with the lower growth inhibition kinetics exhibited by* L. monocytogenes* IMR L55 and a slightly longer exposure time compared to the flower extract for total inactivation. A significant growth kinetic reduction of 4.5 log CFU/mL was observed at pH 4 culminating with total inactivation after 20 h (Figure 2(a)), 7.5 log CFU/mL at pH 6 after 12 h (Figure 2(b)), and 8 log CFU/mL at pH 7 after 4 h (Figure 2(c)). At pH 8, the growth kinetic obtained showed that *L. monocytogenes* IMR L55 was not significantly affected by both extracts even after 24 h incubation (Figure 2(d)).

**Figure 3:**
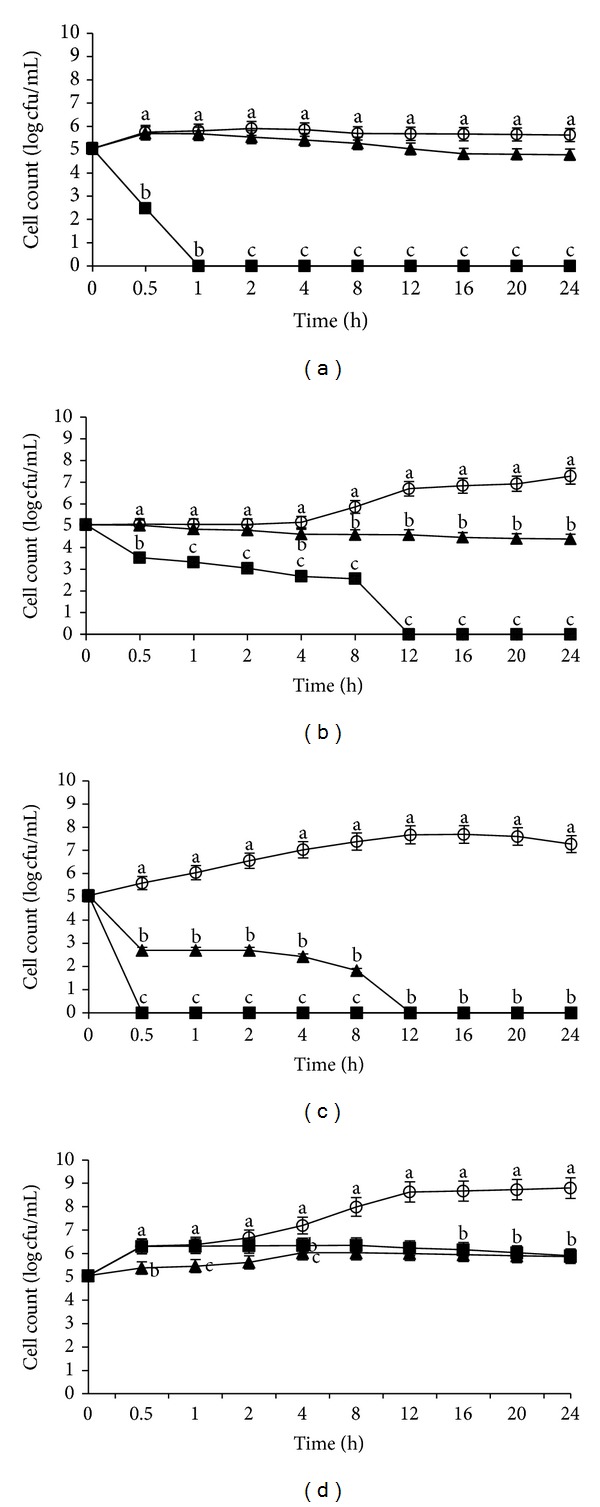
Effects of *Melastoma malabathricum* Linn. extracts on the growth of *Listeria monocytogenes *IMR L55. *L. monocytogenes* IMR L55 was inoculated at various pH values containing 100 mg/mL extracts; (a) pH 4; (b) pH 6; (c) pH 7; (d) pH 8. Cultures were incubated at 25°C. (○) control; (■) flower; (▲) fruit. The values represent the mean ± S.D. of triplicate preformed test for three times repeated. Different letters above each line indicate significant difference between means (*P* < 0.05) within the same inoculation time.

 On the other hand, different growth patterns of *L. monocytogenes* IMR L55 were obtained at 25°C for the same pHs as above (Figure 3). The significant bactericidal effect of the crude flower extract as observed on the reduction of growth kinetic of *L. monocytogenes* IMR L55 at pH 4 was 6 log CFU/mL with total inactivation after 1 h (Figure 3(a)) and 5.5 log CFU/mL at pH 7 with complete inactivation after 30 min (Figure 3(c)). Compared to pH 6 (Figure 3(b)), *L. monocytogenes* IMR L55 had a 7.5 log CFU/mL growth reduction and complete inactivation after 12 h exposure to the crude flower extract. Similar to the earlier findings in this study, longer exposure time was required before significant bactericidal effect was seen on *L. monocytogenes* IMR L55 exposed to the crude fruit extract. It was found that at pH 4 and pH 6, there were growth reductions of 1 log CFU/mL (Figure 3(a)) and 3.0 log CFU/mL (Figure 3(b)), respectively, and a complete inactivation was achieved only after 24 h incubation. However, at pH 7 (Figure 3(c)), a 7.5 log CFU/mL reduction was recorded and complete inactivation achieved after 12 h. Likewise as shown in Figure 2, both crude extracts at pH 8 (Figure 3(d)) did not show any significant bactericidal activity during the 24 h exposure period.

**Figure 4:**
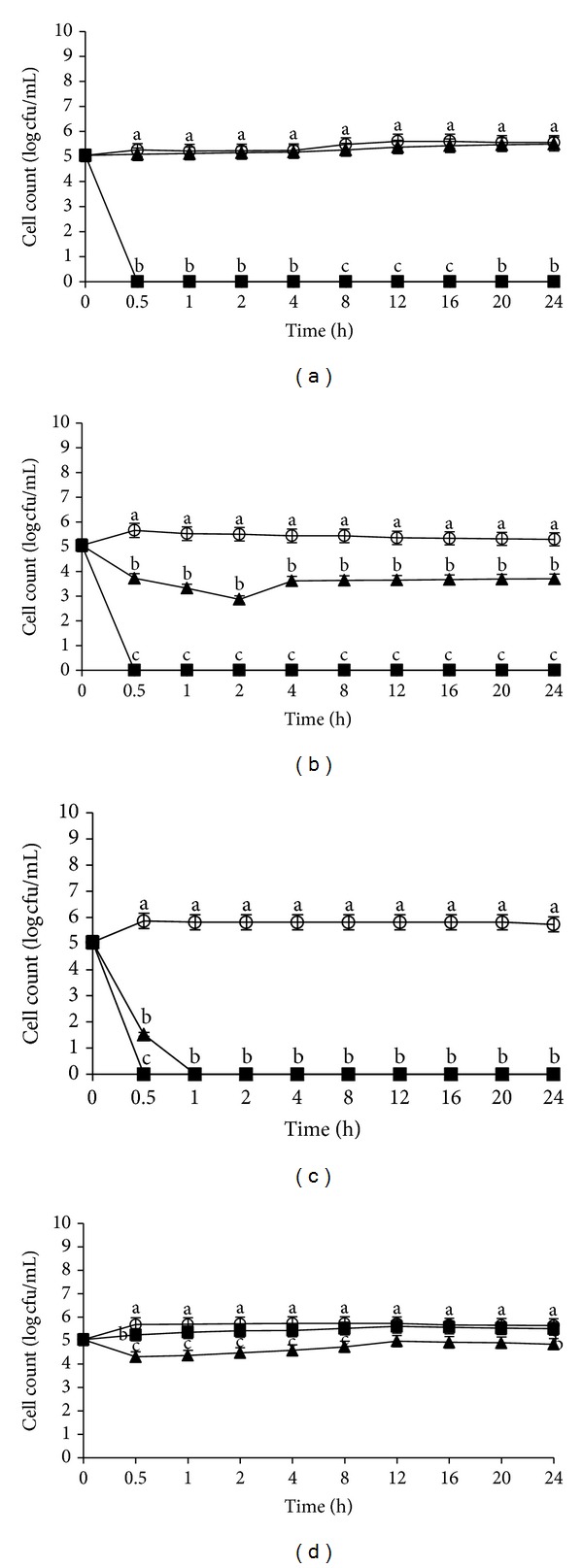
Effects of *Melastoma malabathricum* Linn. extracts on the growth of *Listeria monocytogenes *IMR L55. *L. monocytogenes* IMR L55 was inoculated at various pH values containing 100 mg/mL extracts; (a) pH 4; (b) pH 6; (c) pH 7; (d) pH 8. Cultures were incubated at 4°C. (○) control; (■) flower; (▲) fruit. The values represent the mean ± S.D. of triplicate preformed test for three times repeated. Different letters above each line indicate significant difference between means (*P* < 0.05) within the same inoculation time.

 After 30 min of exposure with crude flower extract at 4°C, *L. monocytogenes* IMR L55 was significantly inactivated with a reduction of 5.0 log CFU/mL at pHs 4, 6, and 7 (Figures 4(a), 4(b), and 4(c), resp.). Exposure to the crude fruit extract at pH 6 showed a 1.5 log CFU/mL reduction after 24 h incubation (Figure 4(b)), while at pH 4, *L. monocytogenes* IMR L55 was not significantly affected when exposed to the crude fruit extract compared with the control (Figure 4(a)). Unlike earlier results of higher incubation temperature, the growth kinetic of *L. monocytogenes* IMR L55, exposed to crude fruit extract, had a higher growth reduction value (5.5 log CFU/mL) with complete inactivation after 1 h incubation at pH 7 (Figure 4(c)). In parallel to all earlier results, *L. monocytogenes* IMR L55 was unaffected significantly when exposed to either the crude flower or fruit extracts even after 24 h when cultured at pH 8 (Figure 4(d)). 

**Figure 5:**
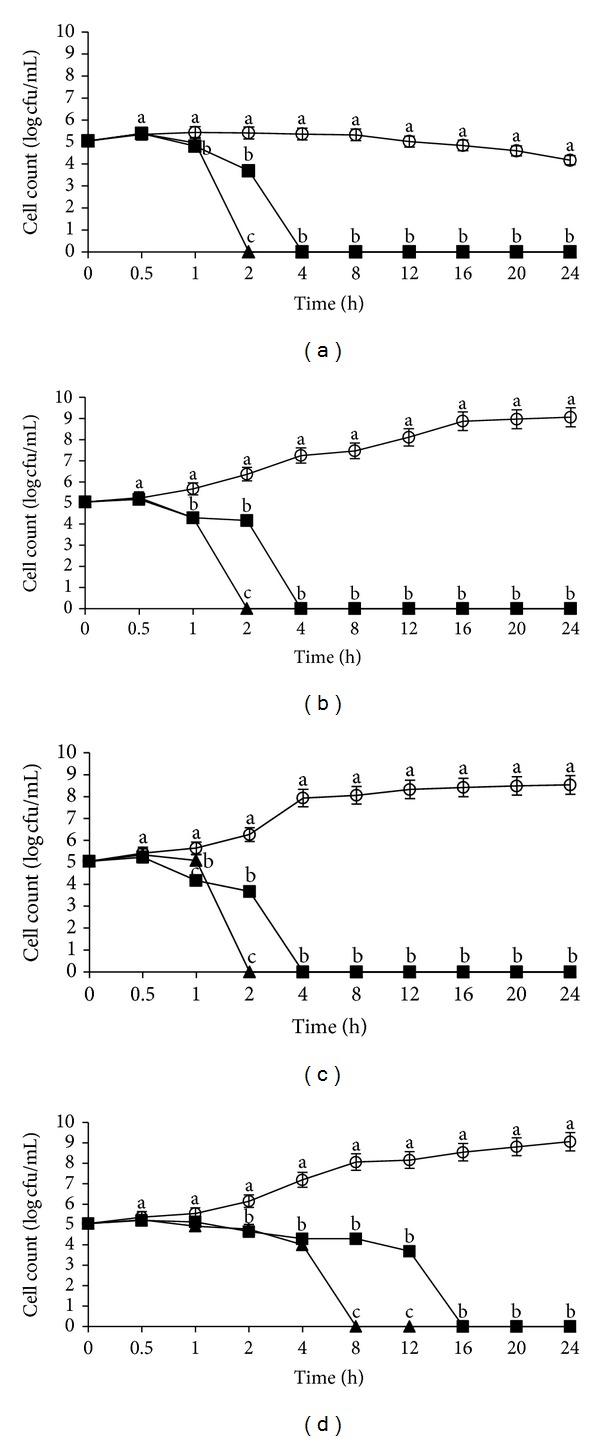
Effects of *Melastoma malabathricum* Linn. extracts on the growth *of Staphylococcus aureus *IMR S244. *S. aureus* IMR S244 was inoculated at various pH values containing 100 mg/mL extracts; (a) pH 4; (b) pH 6; (c) pH 7; (d) pH 8. Cultures were incubated at 37°C. (○) control; (■) flower; (▲) fruit. The values represent the mean ± S.D. of triplicate preformed test for three times repeated. Different letters above each line indicate significant difference between means (*P* < 0.05) within the same inoculation time.

 Unlike* L. monocytogenes* IMR L55, *Staphylococcus aureus *IMR S244 was found to be equally sensitive to both crude extracts. Interestingly, complete inactivation was observed generally to be after a longer exposure period (longer for flower extract compared to fruit extract). The growth profiles of *S. aureus *IMR S244 at 37°C demonstrated significant bactericidal effect with 4 to 6.0 log CFU/mL reduction after 2 h exposure to the crude fruit extract when compared with the control at pHs 4, 6, and 7 (Figures 5(a), 5(b), and 5(c)) while at pH 8, *S. aureus *IMR S244 was completely inactivated with 7 log CFU/mL reduction after 8 h of exposure to the crude fruit extract (Figure 5(d)). Likewise, exposure to crude flower extract also revealed significant complete inactivation after 4 h of exposure coupling with a reduction of 5.0 log CFU/mL at pH 4 (Figure 5(a)), 7.0 log CFU/mL at pH 6 (Figure 5(b)), and 8.0 log CFU/mL at pH 7 (Figure 5(c)). Again, unlike *L. monocytogenes* IMR L55, significant bactericidal effect was observed for *S. aureus *IMR S244 at pH 8 after 16 h exposure to the crude flower extract with a 8.5 log CFU/mL reduction (Figure 5(d)). 

**Figure 6:**
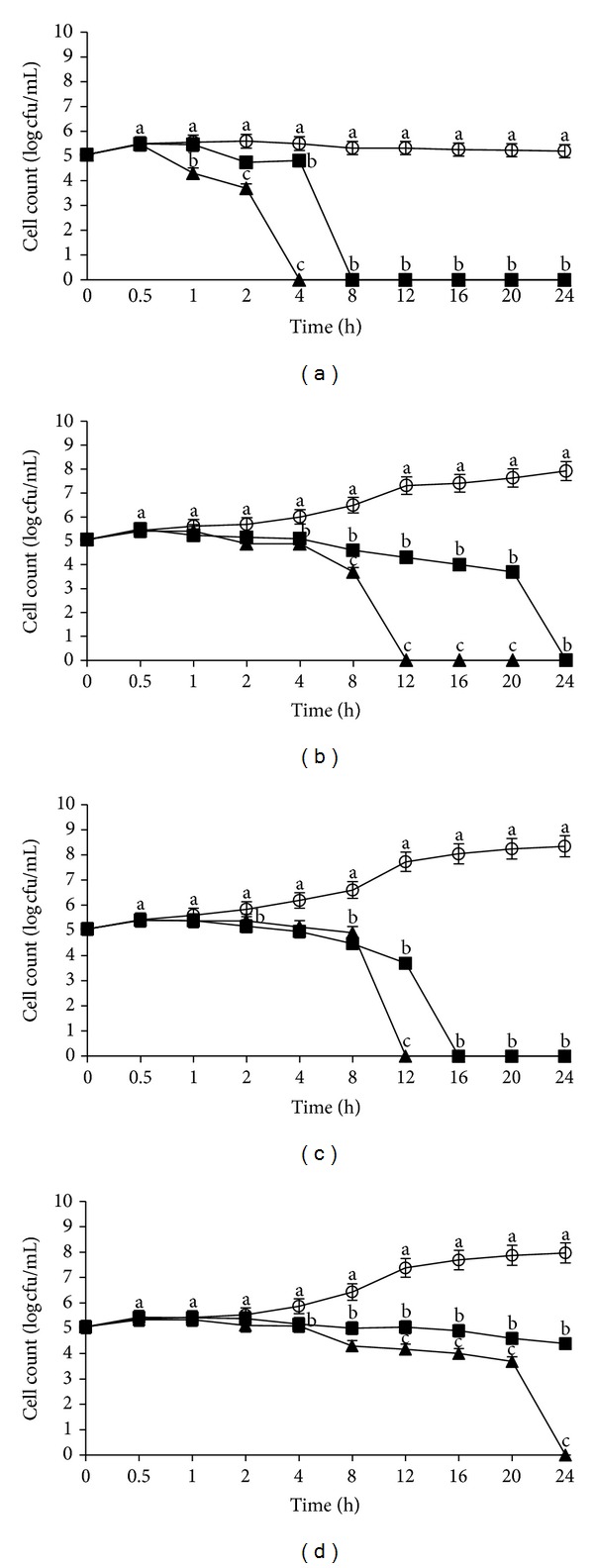
Effects of *Melastoma malabathricum* Linn. extracts on the growth *of Staphylococcus aureus *IMR S244. *S. aureus* IMR S244 was inoculated at various pH values containing 100 mg/mL extracts; (a) pH 4; (b) pH 6; (c) pH 7; (d) pH 8. Cultures were incubated at 25°C. (○) control; (■) flower; (▲) fruit. The values represent the mean ± S.D. of triplicate preformed test for three times repeated. Different letters above each line indicate significant difference between means (*P* < 0.05) within the same inoculation time.

 For 25°C and incubation at pH 4, *S. aureus *IMR S244 was inactivated after 1 h after exposure to the crude fruit extract with a significant growth inhibition kinetic of 6.0 log CFU/mL reduction (Figure 6(a)), while at pHs 6 and 7 (Figures 6(b) and 6(c)), complete inactivation was observed after 12 h with a 7.0 log CFU/mL and 7.5 log CFU/mL reduction, respectively. Again, pH 8 coupled with 25°C significantly inactivated the organisms after 24 h of exposure to the crude fruit extract with a 8.0 log CFU/mL reduction (Figure 6(d)). A similar trend as the crude fruit extract was also observed here for the crude flower extracts at all the pHs tested except pH 8. At pH 4 (Figure 6(a)), *S. aureus *IMR S244 exhibited a 6.0 log CFU/mL reduction with complete inactivation after 8 h exposure. At pH 6 (Figure 6(b)), the count increased to 7.5 log CFU/mL reduction with total wiped out after 24 h exposure, and a 8.0 log CFU/mL reduction at pH 7 (Figure 6(c)) with complete inactivation after 16 h. Interestingly the flower extract at pH 8 (Figure 6(d)) was found to be ineffective against the test organism.

**Figure 7:**
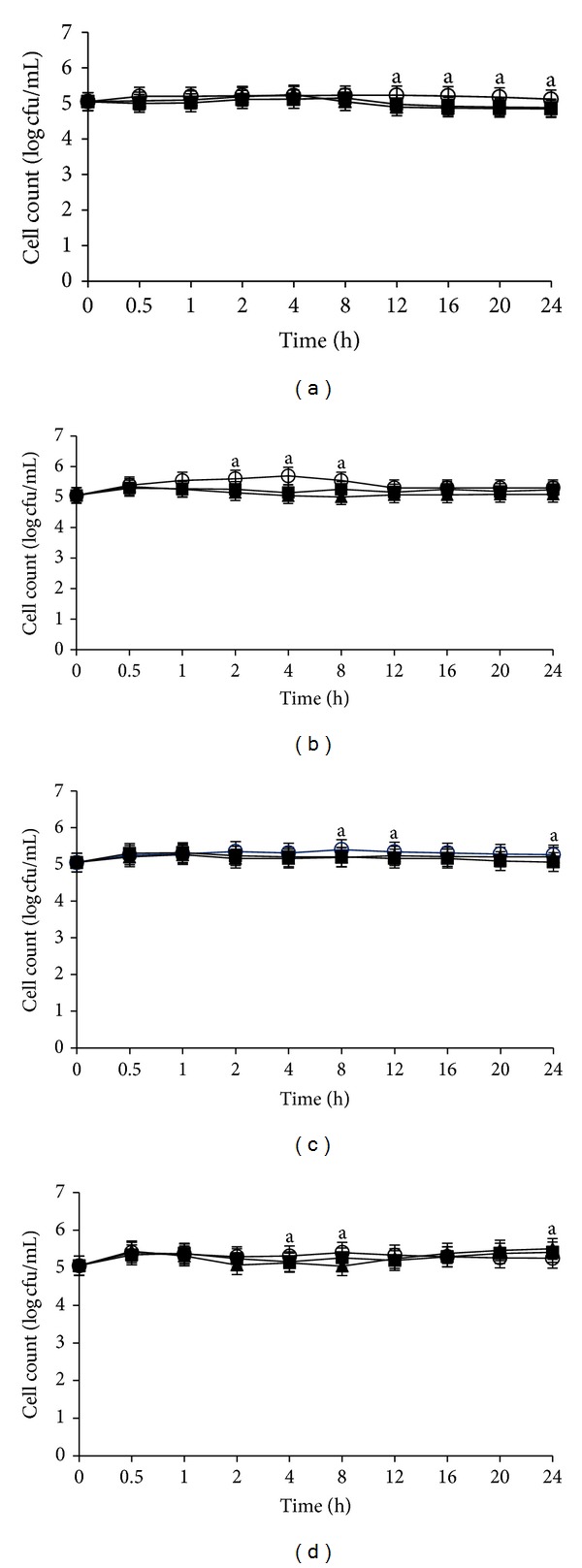
Effects of *Melastoma malabathricum* Linn. extracts on the growth *of Staphylococcus aureus *IMR S44. *S. aureus* IMR S244 was inoculated at various pH values containing 100 mg/mL extracts; (a) pH 4; (b) pH 6; (c) pH 7; (d) pH 8. Cultures were incubated at 4°C. (○) control; (■) flower; (▲) fruit. The values represent the mean ± S.D. of triplicate preformed test for three times repeated. No significant differences (*P* < 0.05) were detected for all the means data within the same inoculation time.

 Overall, the growth kinetic of *S. aureus *IMR S244 was observed to be significantly unaffected at pHs 4, 6, 7, and 8 when exposed to the crude fruit and flower extracts after 24 h incubation when compared with the control at temperature 4°C (Figures 7(a), 7(b), 7(c), and 7(d)).

## 4. Discussion

The MIC and MBC results were consistent with the disc diffusion results showing that Gram-positive bacteria were more sensitive towards the crude flower and fruit extracts. Cushnie and Lamb [22] reported that certain groups of flavonoid compounds exhibited greater inhibition effect on Gram-positive bacteria compared to Gram-negative bacteria. Previous studies on other plant species of Melastomataceae [23–25] also had similar findings. The higher sensitivity reaction by Gram-positive bacteria could be due to the significant differences in the cell wall structure and outer membrane compositions of Gram-positive and Gram-negative bacteria [26]. Gram-negative bacteria possess an outer membrane and a unique periplasmic space not found in Gram-positive bacteria [27, 28]. The resistance of the Gram-negative bacteria towards antibacterial substance could be related to the hydrophilic surface of their outer membrane, as represented by the lipopolysaccharide molecules, which pose as a barrier to the penetration of numerous antimicrobial molecules. Gram-positive bacteria do not have such outer membrane and cell wall structures. Antibacterial substances can easily destroy the bacterial cell wall and cytoplasmic membrane, causing a leakage from the cytoplasm [29]. Mendonça [30] reported that the mechanisms of antimicrobial resistant are dependent on the types of microorganism under consideration as they are related to the bacterial cell structure and the target sites of the microorganism. 

Again, the overall results revealed that the flower extract exhibited more potent activity against all bacteria tested in this study compared to the fruit extract. This could also be attributed to the presence of some different active compounds in the flower and fruit extracts. Susanti et al. [8] reported that the ethyl acetate extract of *M. malabathricum* Linn. flower contained three different compounds such as kaempferol-3-O-*β*-D-glucoside, kaempferol, and naringenin while ethyl acetate extract of the fruit contained betulinic acid. Jofrry et al. [9] discovered active compounds of malvidin-3,5-diglucoside in the *M. malabathricum* Linn. flowers while cyanidin-3-glucoside and cyanidin-3,5-diglucoside were found in the fruits. All those previous studies confirmed that the flower and fruit extracts of *M. malabathricum* Linn. contain different flavonoids compounds. According to Cushnie and Lamb [22], the different structural features of flavonoids may target different components and function of a bacterial cell. They also mentioned that the structural features are necessary for the flavonoids to gain proximity to or uptake into the bacteria cell. Ho et al. [31] reported that the antimicrobial compounds such as flavonoids compounds found in *Orthosiphon stamineus *Benth. extract contributed to the bactericidal activities in the bacterial cells. The published information on *M. malabathricum* Linn. [22, 32] supported the suggestions that the flavonoid compounds present in the flower and fruit extracts of *M. malabathricum* Linn. might have contributed significantly to the antibacterial activity effects observed in this study. 

The results obtained in this study revealed that the bactericidal activities of *M. malabathricum *Linn. crude flower and fruit extracts against *L. monocytogenes* IMR L55 and *S. aureus* IMR S244 were pH and temperature dependent. The growth kinetic of *L. monocytogenes* IMR L55 showed more sensitivity towards the crude flower extract compared to the crude fruit extract at pHs 4, 6, and 7 for all temperature tested (37°C, 25°C, and 4°C), while, at pH 8, *L. monocytogenes* IMR L55 was found to be unaffected when exposed to both crude flower and fruit extracts at all temperatures tested. In contrast to *L. monocytogenes* IMR L55, the growth kinetic of *S. aureus* IMR S244 showed more sensitivity towards the crude fruit extract compared to the crude flower extract at pHs 4, 6, 7 and 8 for 37°C and 25°C. Overall, the growth of *L. monocytogenes* IMR L55 and *S. aureus* IMR S244 was strongly inhibited at pHs 4, 6, and 7, and only slightly at pH 8. These findings were consistent with the observations made by Castañeda-Ovando et al. [33] that anthocyanin compounds were stable in pH values lower than 8. They found that at pH values higher than 7, degradation reaction occurs in the side chains of the anthocyanin compounds. The presence of additional hydroxyl or methoxyl group at the B ring also affects the anthocyanidins stability. Rhodes [34] reported that the hydroxyl groups of flavonoids compounds enhanced inhibitory activities, while methoxy group may increase or decrease the inhibitory activity. They further suggested that the B ring of the flavonoids may play a role in the intercalation or hydrogen bonding with the stacking of nucleic acid bases and, hence, causing inhibitory actions on DNA and RNA synthases in *Proteus vulgaris* and *Staphylococcus aureus*. 

In addition, the results which showed that *S. aureus* was more sensitive to crude fruit extract compared to *L. monocytogenes*, are consistent with other studies that revealed that extracts from the berries fruits (cranberry, cloudberry, and raspberry) containing phenolic compound such as ellagitannin were highly efficient against *S. aureus* but not *L. monocytogenes* [32, 35]. However, Wen et al. [36] found that phenolic acids, such as hydroxycinnamic acids, had bactericidal and bacteriostatic effects against several strains of *L. monocytogenes*. The nature of the antimicrobial effect was contingent upon the medium's pH. Friedman and Jürgens [37] demonstrated that antilisterial effect of caffeic chlorogenic was greater at pH 6.5 than at pH 5.5. Likewise, our study found that antilisterial effect of the crude fruit extract was greater at pH 7 compared to pH 6 at all temperatures tested. It was previously reported that the medium's pH also affected the antimicrobial activity of caffeic, p-coumaric, and ferulic acids towards *S. aureus* and the inhibition on this bacteria was shown to increase as the pH of the bacterial growth medium decreased from pH 7.0 to pH 5.0 [35]. Parallel to the aforementioned findings, in our study *S. aureus* IMR S244 was more sensitive towards the crude fruit extract at lower pH compared to higher pH, especially at 25°C and 37°C. Thus, the medium's pH may have in general great impact on the antimicrobial activity of various phenolic compounds, and there may be complex interactions between pH of the growth media and antimicrobial effects of the berry phenolics varying in different bacterial species and in different phenolic compounds. In contrast, the crude flower extracts showed significant (*P* < 0.05) inhibition of *L. monocytogenes *and *S. aureus *with the former being more sensitive. These findings were consistent with previous studies that flower extracts from *Mentha pulegium* [38],* Teucrium montbretii *subsp. *pamphylicum* [39], and *Plantago major* [40] showed greater inhibition activity against *L. monocytogenes *compared to *S. aureus*.

According to Janna et al. [17], *M. malabathricum* Linn. flower extract showed higher degradation levels on coloured pigments when stored at higher temperature (31°C) compared to lower temperature (25°C) after 26 days. They also found that anthocyanins concentration in the crude extract decreased and the colour faded at higher pHs. So, it was recommended that the suitable storage condition for *M. malabathricum* Linn. flower extract for coloured anthocyanin pigments is in acidic condition (pHs 0.5 and 1.0) and at low temperature (4°C). 

The overall implication from the results obtained in this study is that susceptibility of *L. monocytogenes* to the extracts is very much dependent on the temperature and pH that have significant effects on the optimal growth of the organism and stability of the extracts. The temperature effects on the antibacterial activity showed that *L. monocytogenes* IMR L55 was sensitive to both extracts when cultured at 4°C, 25°C, and 37°C for all the pHs tested. Sensitivity at 4°C could be attributed to the low growth rate of the bacterial population and the highly stable condition of the components in the crude extracts. *L. monocytogenes* was reported to be able to survive between 2°C to 45°C and pH 4 to 9 [41]. In addition, *L. monocytogenes* was found to exhibit sensitivity towards medicinal plants extracts and essential oils with a decreased population growth at 4°C and 25°C within 2 to 4 hours incubation [42]. 

However, different inhibitory effects were found on *S. aureus *IMR S244 which was inhibited by both extracts at 25°C and 37°C for all pHs tested in this study. No significant activity was detected at 4°C. Brener et al. [43] reported that *S. aureus* grew well between 7 and 47°C at pHs 4 to 9. Temperature might be one of the key factors in ensuring stability of the anthocyanins, and these findings were similar to previous study by Mori et al. [44]. 

Overall, *L. monocytogenes* IMR L55 is more sensitive to the flower extract while *S. aureus *IMR S244 is more sensitive to the fruit extract for all pHs and temperatures except 4°C. These results demonstrated that different bacterial species exhibited different sensitivities toward different active compounds in an extract preparation.

## 5. Conclusion

In conclusion, the results of our study showed that the Gram-positive bacteria were more sensitive towards the crude flower and fruit extracts of *M. malabathricum* Linn. compared to the Gram-negative bacteria. *L. monocytogenes *IMR L55 was more sensitive to the crude flower extract at pHs 4, 6, and 7 for all temperatures (4°C, 25°C, and 37°C) tested, while *S. aureus* IMR S244 was more sensitive to the crude fruit extract at 25°C and 37°C and at pHs (4, 6, 7, and 8). In future investigations, bioguided fractionation of both crude extracts is highly recommended to identify the bioactive compounds responsible for the antimicrobial activity. This plant species could prove to be a natural source for the discoveries of alternative medicines in the future. The use of these plant extracts over the years by the local people might be indicative of their safety for the claimed uses. However, further analysis is required to assess the efficacy of the extracts when applied in medicinal treatments, and their toxicity to human needs to be further explored prior to recommendation for alternative medicinal applications. In addition, factors such as temperatures and pHs should be considered as major concerns when the extracts are considered for practical application in alternative medicine as natural products.

## Figures and Tables

**Table 1:** MIC and MBC (mg/mL) of flower and fruit extracts of* Melastoma malabathricum* L.

Bacteria strain	Flower extract (mg/mL)		Fruit extract (mg/mL)	
	MIC	MBC	MIC	MBC
*Listeria monocytogenes* IMR L55	12.5 ± 0.0	100.0 ± 0.0	12.5 ± 0.0	100.0 ± 0.0
*Staphylococcus aureus* IMR S244	100.0 ± 0.0	100.0 ± 0.0	100.0 ± 0.0	100.0 ± 0.0
*Escherichia coli *IMR E30	N.D.	N.D.	N.D.	N.D.
*Salmonella typhimurium* IMR S100	N.D.	N.D.	N.D.	N.D.

Results are the means of MIC and MBC values followed by the standard deviations. All readings are carried out in triplicates. N.D.: no activity detected.
